# Nitrofurantoin Failure in Elderly Men: A Retrospective Observational Study

**DOI:** 10.3390/antibiotics9050211

**Published:** 2020-04-27

**Authors:** Ilse Wolterink, Theo Verheij, Tamara Platteel, Ann van den Bruel, Arjen Stam, Alma van de Pol

**Affiliations:** 1 Julius Center for Health Sciences and Primary Care, University Medical Center Utrecht, Utrecht University, P.O. Box 85500, Universiteitsweg 100, 3508 GA Utrecht, The Netherlands; T.J.M.Verheij@umcutrecht.nl (T.V.); T.N.Platteel-3@umcutrecht.nl (T.P.); A.C.vandePol-11@umcutrecht.nl (A.v.d.P.); 2 Academisch Centrum voor Huisartsgeneeskunde, KU Leuven, Kapucijnenvoer 33 blok J, 3000 Leuven, Belgium; ann.vandenbruel@kuleuven.be; 3 Department of Medical Microbiology, University Medical Center Utrecht, Heidelberglaan 100, 3584 CX Utrecht, The Netherlands; A.J.Stam-2@umcutrecht.nl; 4 Saltro Diagnostic Center for Primary Care, Mississippidreef 83, 3565 CE Utrecht, The Netherlands

**Keywords:** nitrofurantoin, urinary tract infections, treatment failure, anti-bacterial agents, primary care, elderly

## Abstract

Urinary tract infections in the elderly are common. Treatment with nitrofurantoin in men may not be sufficient if concomitant tissue involvement is present, resulting in treatment failure. The aim of this study is to determine the prevalence of nitrofurantoin failure in the elderly, and to assess the effect of gender and age. A retrospective observational study was conducted using a Dutch general practice medical record database of 21,789 men and 26,622 women aged 65 years or older in 2015. First, nitrofurantoin prescriptions in 2015 were analyzed. Nitrofurantoin failure (subsequent prescription of antibiotic within 30 days) for men, women, and different age categories were compared. The effect of age and gender was assessed using multivariate logistic regression. A total of 3537 patients had a first nitrofurantoin prescription in 2015; 506 men and 3031 women. Overall, 584 patients (17%) experienced nitrofurantoin failure; 135 (27%) men and 449 (15%) women. Male gender (odds ratio (OR) = 2.09, 95% confidence interval (CI) 1.68–2.61) and age (OR = 1.02, 95% CI 1.01–1.03) was associated with higher treatment failure. Our findings indicate that in a substantial number of elderly men, nitrofurantoin might not be the appropriate treatment. Nitrofurantoin, as a first choice in elderly men with urinary tract infections, should be reconsidered.

## 1. Introduction

Elderly people have an increased risk of urinary tract infections (UTIs). In men, the incidence of UTI increases with age, from 5 per 1000 patients per year in younger patients, up to 80 per 1000 patients at the age of 80 years [1]. Comparative trials of antimicrobial treatment of UTIs in men are scarce, and the approach to management is mainly based on indirect evidence from trials in women.

Cystitis in men is associated with a higher risk of complications than cystitis in women. Older men, in particular, may be at risk for significant morbidity (i.e., urosepsis, mortality) in case of nitrofurantoin failure [2]. The guideline of the European Association of Urology (EAU) considers all UTIs in men to be complicated [3]. The guideline states that uncomplicated cystitis without involvement of the prostate is uncommon in men. For that reason, the guidelines recommend one to send a urine sample for culture and sensitivity testing in every man with a UTI before the start of antibiotic treatment, whereas women are usually treated empirically [1,3,4]. The guidelines in general also recommend that treatment should last longer in men (mostly 7 days) than in women (either 3 or 5 days). The choice of first-line antibiotic differs between guidelines: The National Institute for Health and Care Excellence (NICE) and the Dutch guidelines recommend nitrofurantoin, whereas the EAU guideline recommends trimethoprim/sulfamethoxazole [1,3,4]. The latter is also considered as an option by NICE. The treatment of UTI in men with nitrofurantoin may not be sufficient if concomitant tissue involvement is present, resulting in treatment failure, which puts elderly men at risk of severe complications.

The aim of this study was to explore whether treatment failure occurs in routine practice, using medical records from a large general practice network to determine the prevalence of nitrofurantoin failure in elderly men and women, and to assess the effect of age on nitrofurantoin failure.

## 2. Results

A total of 48,411 patients in the Julius General Practitioners’ Network (JGPN) database were 65 years or older in 2015; 21,789 (45%) men and 26,622 women (55%). The mean age of all patients was 74 years, with mean ages in men and women of 73 and 75 years, respectively.

A total of 29,261 antibiotic prescriptions for any cause were prescribed to 12,427 patients, i.e., 26% of the population under study received at least one antibiotic in 2015. Overall, 4712 (22%) of men and 7715 (29%) of women received at least one antibiotic prescription. Nitrofurantoin was the most commonly prescribed antibiotic, with 7060 (24%) of all antibiotic prescriptions; 815 (2.7%) prescriptions in men and 6245 (21%) prescriptions in women.

A total of 506 men (2% of all men, 11% of men with an antibiotic prescription) were prescribed nitrofurantoin at least once in 2015, compared to 3031 women (11% of all women, 39% of women with at least one antibiotic prescription). Of the 506 men receiving a first nitrofurantoin prescription, 135 (27%) had nitrofurantoin failure (Table 1). Of the 3031 women receiving a first nitrofurantoin prescription, 449 (15%) had nitrofurantoin failure. Male gender was associated with a higher risk of nitrofurantoin failure (OR = 2.09, 95% CI 1.68–2.61), as well as age (in years) (OR = 1.02, 95% CI 1.01–1.03).

In men, ciprofloxacin was most commonly prescribed following the first course of nitrofurantoin, whereas in women, this was fosfomycin (Table 2).

## 3. Discussion

In our retrospective observational study using a Dutch general practice medical record database containing the prescription data of 21,789 men and 26,622 women aged 65 years or older, nitrofurantoin was the most commonly prescribed antibiotic. A total of 3537 patients had a first nitrofurantoin prescription in 2015, of which 584 patients (17%) experienced nitrofurantoin failure; 135 (27%) men and 449 (15%) women. Nitrofurantoin failure prevalence increased with age.

The current study has a number of limitations. First, physicians’ diagnosis code of UTI and data on hospital admissions were not available from our database. As a proxy for UTI, we selected prescriptions of nitrofurantoin, because this is rarely, if ever, prescribed for anything other than suspicion of UTI. However, validity for prescribing nitrofurantoin and specific symptoms such as fever that indicate tissue involvement could not be investigated. Prophylactic nitrofurantoin prescriptions for recurrent UTIs, and nitrofurantoin prescriptions that were prescribed preventatively in case the patients’ symptoms would worsen, but in which patients eventually did not need or use the medication, were not excluded from the analysis. Additionally, data considering antibiotics prescribed at out-of-hours services were not available for analysis. Although the overall population was large, outcome numbers for subanalysis for specific age categories were smaller, making results more susceptible to random variations.

The current study also has several strengths. A very large study population was used, increasing the power of the statistical analyses and reliability of the prevalence rates. This is further supported by the large amount of antibiotic prescriptions. The results are highly illustrative for the degree in which elderly cope with UTI, and the rate in which treatment failure occurs.

Although other studies looking at nitrofurantoin treatment failure exist, this is the first study to investigate treatment failure in older adults specifically, only including antibiotics that are used for UTI and with a 30-day follow-up. The Dutch guideline reports a previous unpublished study by the Netherlands institute for health services research Nivel, that also estimated nitrofurantoin treatment failure at 27% in men of all ages [1]. However, their treatment failure definition also included antibiotics that were likely not prescribed for UTIs, and they considered a 60-day period. Another recent study on outpatient antibiotic use in the Netherlands showed 5.4% of patients treated with nitrofurantoin were prescribed a different antibiotic within 3 days across all ages [5]. This much lower treatment failure rate can be explained by the 3-day treatment failure period, as opposed to the 30-day period in our study. Finally, Goettsch et al. (2004) found a treatment failure of 14% in women 15–65 years, also increasing with age [6].

In our study, the choice of the second antibiotic treatment was consistent with Dutch guidelines for women, as most women are prescribed either fosfomycin or trimethoprim (48% resp. 21%). In men, ciprofloxacin accounts for more than half of second antibiotic prescriptions after nitrofurantoin, followed by trimethoprim (17%). In the current Dutch guidelines, ciprofloxacin is recommended if there are signs of tissue involvement (i.e., prostatitis and pyelonephritis).

Possible tissue involvement in UTIs in men may explain the higher failure of nitrofurantoin in men than in women, and why ciprofloxacin was selected in half of the men as second prescription. This hypothesis is supported by another study which showed that in more than 90% of men with febrile UTIs, prostate infection was involved [7]. However, ciprofloxacin, as an alternative for nitrofurantoin in case of treatment failure, may not be suitable, due to the higher resistance rate compared to nitrofurantoin (10% resp. 3%) [8], as well as the possibility of serious side effects of fluoroquinolones [9]. This demonstrates the importance of a urine culture in risk groups and taking antibiotic resistance and possible side effects into account when searching for an alternative for nitrofurantoin.

In our study, we found that in more than a quarter of the men receiving nitrofurantoin, different antibiotic prescriptions for UTIs were necessary within 30 days. This indicates that in a substantial portion of the elderly men, nitrofurantoin might not be the appropriate treatment. More comparative studies for the efficacy of antibiotic treatment, specifically of nitrofurantoin, for UTIs in elderly men are needed. Due to the high prevalence of treatment failure and possible risk of severe morbidity, nitrofurantoin, as a first choice in elderly men with UTIs, should be reconsidered.

## 4. Materials and Methods

### 4.1. Cohort

This is an observational retrospective cohort study using routinely collected data. Data were obtained from the JGPN database. This database contains anonymized electronic medical records from 64 general practices, including 371,028 patients in The Netherlands, and is representative of the Dutch population [10]. We selected all patients who were registered in one of the participating practices on 1 January 2015 and were 65 years or older in 2015 (*n* = 48,411). Age, gender and antibiotic prescriptions with corresponding dates were available for analysis. Prescriptions were recorded using the Anatomical Therapeutic Chemical (ATC) Classification System.

### 4.2. Data Analysis

We analyzed all first nitrofurantoin prescriptions in 2015 of the cohort of 48,411 selected patients, the date of prescription being the index date. Probable failure of nitrofurantoin was defined as a prescription within 30 days after the index date for a different antibiotic that is recommended for UTIs, i.e., fluoroquinolones (ciprofloxacin, levofloxacin, norfloxacin, ofloxacin), fosfomycin, trimethoprim, amoxicillin/clavulanic acid, and trimethoprim/sulfamethoxazole. Other antibiotics such as amoxicillin, azithromycin, doxycycline or flucloxacillin were excluded from this definition, as these are likely to be prescribed for reasons other than UTIs. A second prescription of nitrofurantoin was excluded from the definition, as this was assumed to be a prolongation of the initial treatment, a prophylactic prescription, or a new episode of an uncomplicated UTI. Multivariate logistic regression was performed to assess the effects of age and gender on treatment failure. For data-analysis, Statistical Package for the Social Sciences version 25.0 (Armonk, NY, USA) was used.

### 4.3. Ethics

Research in the JGPN database is observational, and data are generated during routine care; enlisted patients are not approached individually for participation, but are adequately informed at the start of their enrolment, offering them the option to opt out [10]. The Medical Ethics Committees in the Netherlands do not consider such research as subject to the Dutch law on medical research involving human subjects. Data were handled in adherence with Dutch privacy legislation.

## 5. Conclusions

This retrospective observational study analyzed the prevalence of nitrofurantoin failure in UTI in elderly men and women. Our findings indicate that treatment failure occurred in a substantial number of elderly men, and nitrofurantoin might not be the appropriate treatment. Nitrofurantoin, as a first choice in elderly men with UTIs, should be reconsidered.

## Figures and Tables

**Table 1:** First nitrofurantoin prescription in 2015 and failure prevalence for men and women.

Age (Years)		65–75	76–85	>85	Total
Total	First prescription nitrofurantoin, *n*	1610	1241	686	3537
	Nitrofurantoin failures, *n* (%)	228 (14)	221 (18)	135 (20)	584 (17)
Men	First prescription nitrofurantoin, *n*	208	211	87	506
	Nitrofurantoin failures, *n* (%)	52 (25)	60 (28)	23 (26)	135 (27)
Women	First prescription nitrofurantoin, *n*	1402	1030	599	3031
	Nitrofurantoin failures, *n* (%)	176 (13)	161 (16)	112 (19)	449 (15)

**Table 2:** Distribution prescribed antibiotics within 30 days after first nitrofurantoin prescription in 2015.

Types of Antibiotics	Represcriptions for Men (*n* = 135)	Represcriptions for Women (*n* = 449)
Ciprofloxacin, *n* (%)	71 (53)	77 (17)
Amoxicillin/clavulanic acid, *n* (%)	24 (18)	48 (11)
Trimethoprim, *n* (%)	23 (17)	92 (21)
Trimethoprim/sulfamethoxazole, *n* (%)	8 (6)	7 (2)
Fosfomycin, *n* (%)	5 (4)	215 (48)
Norfloxacin, *n* (%)	4 (3)	9 (2)
Ofloxacin, *n* (%)	0	1 (0.2)

## References

[1] Van Pinxteren B., Knottnerus B.J., Geerlings S.E., Visser H.S., Klinkhamer S., Van der Weele G.M., Verduijn M.M., Opstelten W., Burgers J.S., Van Asselt K.M. (2013). NHG-Standaard Urineweginfecties. Huisarts Wet..

[2] Gharbi M., Drysdale J.H., Lishman H., Goudie R., Molokhia M., Johnson A.P., Holmes A.H., Aylin P. (2019). Antibiotic management of urinary tract infection in elderly patients in primary care and its association with bloodstream infections and all cause mortality: Population based cohort study. BMJ.

[3] Bonat G., Pickard R., Bartoletti R., Bruyère T.C.F., Geerlings S.E., Köves B., Wagenlehner F. (2018). European Association of Urology Guideline on Urological Infections. EAU Annu. Congr..

[4] National Institute for Health and Care Excellence Guideline (2018). Urinary Tract Act Infection (Lower): Antimicrobial Prescribing NG109. NICE.

[5] De Jong L.A.W., Van Der Linden P.D., Roukens M.M.B., Van de Garde E.M.W., Van der Velden A.W., Natsch S. (2019). Consecutive antibiotic use in the outpatient setting: An extensive, longitudinal descriptive analysis of antibiotic dispensing data in the Netherlands. BMC Infect Dis..

[6] Goettsch W.G., Janknegt R., Herings R.M.C. (2004). Increased treatment failure after 3-days’ courses of nitrofurantoin and trimethoprim for urinary tract infections in women: A population-based retrospective cohort study using the PHARMO database. Br. J. Clin. Pharmacol..

[7] Ulleryd P. (2003). Febrile urinary tract infection in men. Int. J. Antimicrob. Agents.

[8] Geerlings S.E., Van Nieuwkoop C., Van Haarst E., Van Buren M., Knottnerus B.J., Stobberingh E.E., De Groot C.J., Prins J.M. (2013). Stichting Werkgroep Antibioticabeleid Guideline for Antimicrobial Therapy of Complicated Urinary Tract Infections in Adults. SWAB.

[9] European Medicines Agency (2018). Disabling and potentially permanent side effects lead to suspension or restrictions of quinolone and fluoroquinolone antibiotics. Eur. Med. Agency.

[10] Smeets H.M., Kortekaas M.F., Rutten F.H., Bots M.L., Van der Kraan W., Daggelders G., Smits-Pelser H., Helsper C.W., Hoes A.W., De Wit N.J. (2018). Routine primary care data for scientific research, quality of care programs and educational purposes: The Julius General Practitioners’ Network (JGPN). BMC Health Serv. Res..

